# Educational differences in cancer incidence, stage at time of diagnosis, and survival in Norway

**DOI:** 10.1007/s43999-025-00075-z

**Published:** 2025-10-03

**Authors:** Kenz Al-Shather, Yngvar Nilssen, Inger Kristin Larsen, Erlend Hem, Berit Horn Bringedal

**Affiliations:** 1 Department of Behavioural Medicine, Institute of Basic Medical Sciences, Faculty of Medicine, University of Oslo, Oslo, Norway; 2 Institute for Studies of the Medical Profession, PO Box 1152, Sentrum, Oslo, NO‑0107 Norway; 3 Department of Registration, Cancer Registry of Norway, Oslo, Norway

**Keywords:** Cancer incidence, Cancer stage at diagnosis, Cancer survival, Social inequality, Health inequality, Norway

## Abstract

**Aim:**

The study investigates differences in cancer incidence, stage, and survival between municipalities with varying levels of education in Norway. It replicates the design of a similar study conducted in Oslo to assess whether similar patterns are present nationwide.

**Method:**

We used aggregated data from the Cancer Registry of Norway (2014–2023) to calculate age-standardized incidence rates, stage at diagnosis, and five-year relative survival for colon, rectal, lung, melanoma, breast, and prostate cancer across municipalities. Municipalities were grouped by educational level (low, medium, high), and outcomes were compared.

**Results:**

The study included 191,213 cases. Age-standardized incidence rates (per 100,000 person-years) differed significantly by education for all cancer types except breast cancer (*p* = 0.70). Melanoma and breast cancer incidence was highest in high-education areas (melanoma: 45.2 vs. 36.9; breast: 135.6 vs. 120.3), while lung cancer was highest in low-education areas (64.4 vs. 56.3). Colon, rectal, and prostate showed smaller but significant differences (colon: 54.7 vs. 54.5, *p* < 0.001; rectal: 24.5 vs. 25.9, *p* < 0.001; prostate: 189.8 vs. 191.5, *p* < 0.001). Low-education areas had the highest proportion of distant metastases for most cancers, with significant variation for lung (*p* = 0.003) and prostate (*p* < 0.001). Mid- or high-education areas more often had localized disease, except melanoma. Low-education areas had lower five-year relative survival, significant only for breast cancer (*p* = 0.037).

**Conclusion:**

Cancer incidence, stage, and survival varied between municipalities grouped by inhabitants’ educational level. Findings align with those of the Oslo study, highlighting consistent education-related disparities in cancer outcomes nationwide.

**Supplementary Information:**

The online version contains supplementary material available at 10.1007/s43999-025-00075-z.

## Introduction

Since 2017, cancer has been the leading cause of death in Norway [1, 2], and the global cancer burden continues to grow [3]. Considering the challenges cancer poses to patients and their families, an effective healthcare system is needed to provide proper support and care. The Norwegian healthcare system is committed to universal access, where tax payments largely finance public health services [4]. Patients contribute with smaller copayments, which cover a range of services, including primary, ambulatory, mental health, and hospital care [4]. Consequently, Norwegian residents can access comprehensive healthcare coverage regardless of socioeconomic background and age. In addition, Norway implemented policy strategies to standardize cancer treatment, including national treatment guidelines, cancer patient pathways, and cancer coordinators in municipalities [1].

Despite efforts to prevent inequalities in cancer care, evidence suggests that social disparities persist in Norway. A recent OECD report on European countries found that groups with lower education were less likely to receive screening, had a higher prevalence of major cancer risk factors, and had higher cancer mortality [5]. Furthermore, numerous studies highlight a higher incidence of several cancer types among those with lower education. Preventable cancers, like lung and cervical cancer, show the most pronounced socioeconomic inequalities [6–8]. Additionally, previous Norwegian studies have demonstrated that educational level may influence how early cancer is diagnosed and treatment for lung and colorectal cancer [9–11]. Reducing socioeconomic inequalities in cancer care is essential for both lowering national cancer mortality rates and advancing health equity across all of Norway.

While substantial research has been conducted on cancer inequalities in Norway, previous studies have often focused only on a few specific cancer sites or examined cancer incidence, stage at diagnosis, and survival independently rather than together [6, 8, 9, 11]. By investigating incidence, stage, and survival by educational level in a single study, we can better understand how educational level influences the entire course of cancer, from diagnosis to survival outcomes. By focusing on data from the most recent decade, we ensure that the findings reflect current trends in cancer care and diagnostics. This can offer insight to help guide public health initiatives and improve cancer care for all patient groups.

In this study, we use education as a measure of socioeconomic status. While other features of municipalities, such as average income, the share of immigrants, and poverty rates, also influence local living conditions relevant to health, educational level is especially relevant in understanding cancer disparities because it often reflects differences in health literacy and risk behaviors [12–18]. Furthermore, unlike income or occupation, educational level tends to remain constant throughout adulthood, which can make it a more reliable measure for long-term studies. In Norway, where social welfare systems moderate income differences [19], educational attainment can be a more effective indicator of health disparities.

This study examines education at the municipal level. While most prior research uses individual-level data, studying municipalities may reveal insights not apparent at the individual level. Municipalities vary in health knowledge, social norms, and quality of primary health services. These differences might affect patient behavior, healthcare access, and cancer outcomes. Although aggregate measures do not replace individual analyses, they may offer valuable insight into contextual influences on cancer outcomes.

A recent study in Oslo examined educational disparities in cancer outcomes at the district level. Districts with higher educational levels had increased incidences of breast, melanoma, and prostate cancers. Districts with lower educational levels had a higher incidence of lung cancer [20]. Residents in low-education areas were more frequently diagnosed at advanced stages. They also had lower five-year survival rates overall [20]. Residents in low-education areas were more frequently diagnosed at advanced stages. They also had lower five-year survival rates overall [20]. These patterns suggest disparities in early detection of cancer and survival outcomes linked to educational differences.

Our study examines educational differences between municipalities. This allows us to compare the results with the findings from Oslo to investigate whether similar patterns exist on a national level. Municipalities were chosen instead of counties because they are responsible for primary health care, public health measures, and welfare services locally in Norway. We therefore assumed that access to health care would be more homogeneous within these units than if we had used counties as the units of analysis.

While the Oslo study provided important insights into disparities in an urban area it is still unclear whether this represents a general pattern, or whether these disparities are specific to Oslo. Oslo’s population and healthcare system may not fully represent the entire country. It has the highest population density in Norway [21] and the most diverse population, with 34.7% of residents being immigrants or born in Norway to immigrant parents [22]. Oslo is also home to some of Norway’s largest and most specialized hospitals [23]. To our knowledge, no recent studies have simultaneously examined cancer incidence, stage at diagnosis, and survival across municipalities with different educational levels in Norway. A national study is therefore needed to determine whether these patterns are consistent across Norway, and this study aims to replicate and expand the Oslo research to a national scale.

## Methods

### Study sample and data sources

This is a registry-based study that included the most common cancer sites diagnosed in the period from 1 January 2014 to 31 December 2023. The cases were identified using the Cancer Registry of Norway (CRN), and the following cancer sites were included: colon (C18), rectum (C19–20), lung (C33–34), melanoma (C43), breast (C50), and prostate (C61) cancer. All cases were identified according to the International Classification of Diseases and Related Health Problems (ICD-10).

Cancer stage at diagnosis was classified into localized, where cancer remains only in the organ it started in; regional, where cancer has grown into nearby organs, structures, or lymph nodes; and distant, indicating the spread of cancer to distant lymph nodes or distant parts of the body; and unknown, where no information about the stage was available. The categories were based on the American National Cancer Institute (NCI) Surveillance, Epidemiology, and End Results (SEER) Summary Staging system. This classification is often used for statistical purposes and facilitates long-term comparisons [24].

In addition to patient and tumor characteristics like date of diagnosis, stage at diagnosis, and date of death, we also obtained patients’ place of residence through the CRN. The CRN was established in 1952 and has since 1953 systematically registered nationwide cancer data. Mandatory reporting by healthcare providers ensures nearly complete data coverage, with a completeness rate of 98.7% for 2019–2023 [25].

Despite the possibility of studying this over a longer timeframe, we chose to restrict the analysis to a ten-year period. A longer period would make interpretations more difficult, since changes in cancer treatments and diagnostics over time influence outcomes in ways that are hard to account for. On the other hand, looking at a shorter period would increase the risk of random year-to-year variations affecting the results. By selecting a ten-year period, we aim to reduce the impact of changes in cancer care while ensuring that the timeframe is long enough to minimize the influence of random variation.

Norway’s population is about 5.6 million [26]. Norway is divided into municipalities, which function as the primary units of local government and are responsible for key services such as primary healthcare, social services, primary and secondary education, and infrastructure, among other things [27]. The municipal structure has significantly changed over the years, with municipalities divided and boundaries adjusted. This study is based on the municipal divisions in Norway as of January 2023. At that time, Norway had 356 municipalities.

The educational level was based on the percentage of municipal residents with more than four years of college or university education. We used this to categorize municipalities into low-, mid-, and high-educational levels, setting the cut-off points at the 33rd and 66th percentiles. In high-education municipalities, more than 6.9% of residents had more than four years of higher education, while in mid-education municipalities, this ranged between 5.2% and 6.9%. In low-education municipalities, less than 5.2% of residents had more than four years of higher education. Data on the educational level in the municipalities for 2023 was obtained from Statistics Norway [28].

### Statistical analysis

Incidence rates were adjusted for age using direct age standardization by applying a weighted average of the rates observed across 18 distinct age brackets (0–4, 5–9, …, 85+). The weights were based on the age distribution of the Norwegian population in 2014. The age-standardized incidence rate for all the cancer types was calculated for the three education areas and is reported as cases per 100,000 person-years. To assess whether incidence rates differed significantly across municipalities based on proportion of high education, we fitted a Poisson regression model with the number of cancer cases as the dependent variable and educational level as the main independent variable. Population size was included as an offset term to account for differences in exposure time across groups. An overall difference in incidence rates between groups was evaluated using a likelihood ratio test. Differences in stage at diagnosis across educational groups were assessed using Pearson’s chi-square test.

Relative survival was estimated using flexible parametric survival models, with restricted cubic splines for the baseline cumulative hazard function, implemented via the stpm3 framework [29]. Expected mortality rates were obtained from national life tables stratified by sex, one-year age group, and calendar year, and were linked to each individual at the time of diagnosis. Relative survival up to five years after diagnosis was predicted for each educational group using post-estimation marginal standardization with the standsurv package, adjusting for age at diagnosis, sex, urban/rural residence, and stage at diagnosis. The independent contribution of educational level to survival differences was assessed by comparing a fully adjusted model (including age, sex, urban/rural residence, stage, and education) to a nested model excluding education. The statistical significance of education was evaluated using a likelihood ratio test based on model fit. All statistical analyses were performed using Stata 18.

## Results

The study included 191,213 cancer cases registered between 1 January 2014 and 31 December 2023. This consists of 31,270 cases of colon cancer (16.4%), 14,034 cases of rectal cancer (7.3%), 33,401 cases of lung cancer (17.5%), 23,910 cases of melanoma (12.5%), 36,826 cases of breast cancer (19.3%), and 51,772 cases of prostate cancer (27.1%).

In Norway, 12.0% of residents over the age of 16 had more than four years of higher education in 2023. Municipal educational levels ranged from 2.7% to 24.1%, with a mean of 6.7%. Urban areas with universities and job opportunities for higher education individuals rank high in education levels. In contrast, less central locations with economies centered around industries that do not require high levels of education rank lower. Table 1 presents the total number of residents, cancer cases, municipalities, and basic demographic characteristics.

**Table 1:** Table presenting the total number of residents, cancer cases, municipalities, and basic demographic characteristics across the three educational categories. Municipalities were grouped by educational level (low, medium, high) based on the percentage of the population with more than four years of higher education: low (≤ 5.2%), mid (5.2–6.9%), high (6.9–24.1%). Population numbers are based on the 2023 mid-year population from the cancer registry of Norway, calculated as the mean of the population size on 1 January 2023 and 1 January 2024

	Municipality group		
	Low	Mid	High
N	20,126 (10.5%)	39,918 (20.9%)	131,169 (68.6%)
ICD10gr			
Colon	3,360 (16.7%)	6,795 (17.0%)	21,115 (16.1%)
Rectum	1,574 (7.8%)	2,983 (7.5%)	9,477 (7.2%)
Lung	4,032 (20.0%)	7,436 (18.6%)	21,933 (16.7%)
Melanoma of the skin	2,092 (10.4%)	4,365 (10.9%)	17,453 (13.3%)
Breast	3,312 (16.5%)	6,978 (17.5%)	26,536 (20.2%)
Prostate	5,756 (28.6%)	11,361 (28.5%)	34,655 (26.4%)
Age at diagnosis	69.3 (12.0)	68.7 (12.3)	67.9 (12.9)
Sex			
Females	8,537 (42.4%)	17,271 (43.3%)	60,786 (46.3%)
Males	11,589 (57.6%)	22,647 (56.7%)	70,383 (53.7%)
Number of municipalities	110	123	123
Urban (%)*			
>=80%	2,516 (12.5%)	9,342 (23.4%)	112,539 (85.8%)
20%-80%	16,353 (81.3%)	29,872 (74.8%)	18,306 (14.0%)
<20%	1,257 (6.2%)	704 (1.8%)	324 (0.2%)
Population	479,065	1,040,108	4,000,421

* Urban classification based on Statistics Norway’s (SSB) definition of urban settlement [52]

### Incidence

Table 2 displays the age-standardized incidence rate per 100,000 person-years in the low-, mid-, and high-education areas. Colon, rectal, lung, melanoma, and prostate cancer all showed statistically significant differences in incidence rates across education groups (*p* < 0.001), while no significant difference was observed for breast cancer (*p* = 0.70).

**Table 2:** Age-standardized incidence rate per 100,000 person-years in the low-, mid-, and high-education areas for all included cancer types (95% confidence interval in parentheses). Municipalities were grouped by educational level (low, medium, high) based on the percentage of the population with more than four years of higher education: low (≤ 5.2%), mid (5.2–6.9%), high (6.9–24.1%). Overall *p*-values for group differences are based on likelihood ratio tests from Poisson regression

	Low-education areas	Mid-education areas	High-education areas	*p*-value
**Colon cancer**	54.5 (52.7–56.4)	56.6 (55.3–58.0)	54.7 (54.0–55.5)	*p* < 0.001
**Rectal cancer**	25.9 (24.6–27.2)	25.2 (24.3–26.3)	24.5 (24.0–25.0)	*p* < 0.001
**Lung cancer**	64.4 (62.4–66.5)	61.0 (59.6–62.4)	56.3 (55.6–57.1)	*p* < 0.001
**Melanoma of the skin**	36.9 (35.3–38.6)	38.1 (37.0–39.3)	45.2 (44.6–45.9)	*p* < 0.001
**Breast cancer**	120.3 (116.1–124.6)	124.0 (121.1–127.0)	135.6 (134.0–137.2)	*p* = 0.70
**Prostate cancer**	191.5 (186.5–196.6)	194.6 (191.0–198.3)	189.8 (187.8–191.9)	*p* < 0.001

Colon cancer rates were similar across educational levels (54.5 for low, 56.6 for mid, and 54.7 for high); rectal cancer rates also show a similar pattern (25.9 for low, 25.2 for mid, and 24.5 for high). Prostate cancer rates were consistently high across all education levels (191.5 for low, 194.6 for mid, and 189.8 for high). For lung cancer, the incidence rate decreases with higher educational levels, showing a negative gradient (64.4 for low, 61.0 for mid, and 56.3 for high). Incidence rates for both melanoma and breast cancer show a positive gradient with increasing education levels. Melanoma incidence rates rise from 36.9 in low-education areas to 38.1 in mid and 45.2 in high. Similarly, breast cancer rates were higher in mid- and high-education areas (120.3 for low, 124.0 for mid, and 135.6 for high).

### Stage at time of diagnosis

**Fig. 1:**
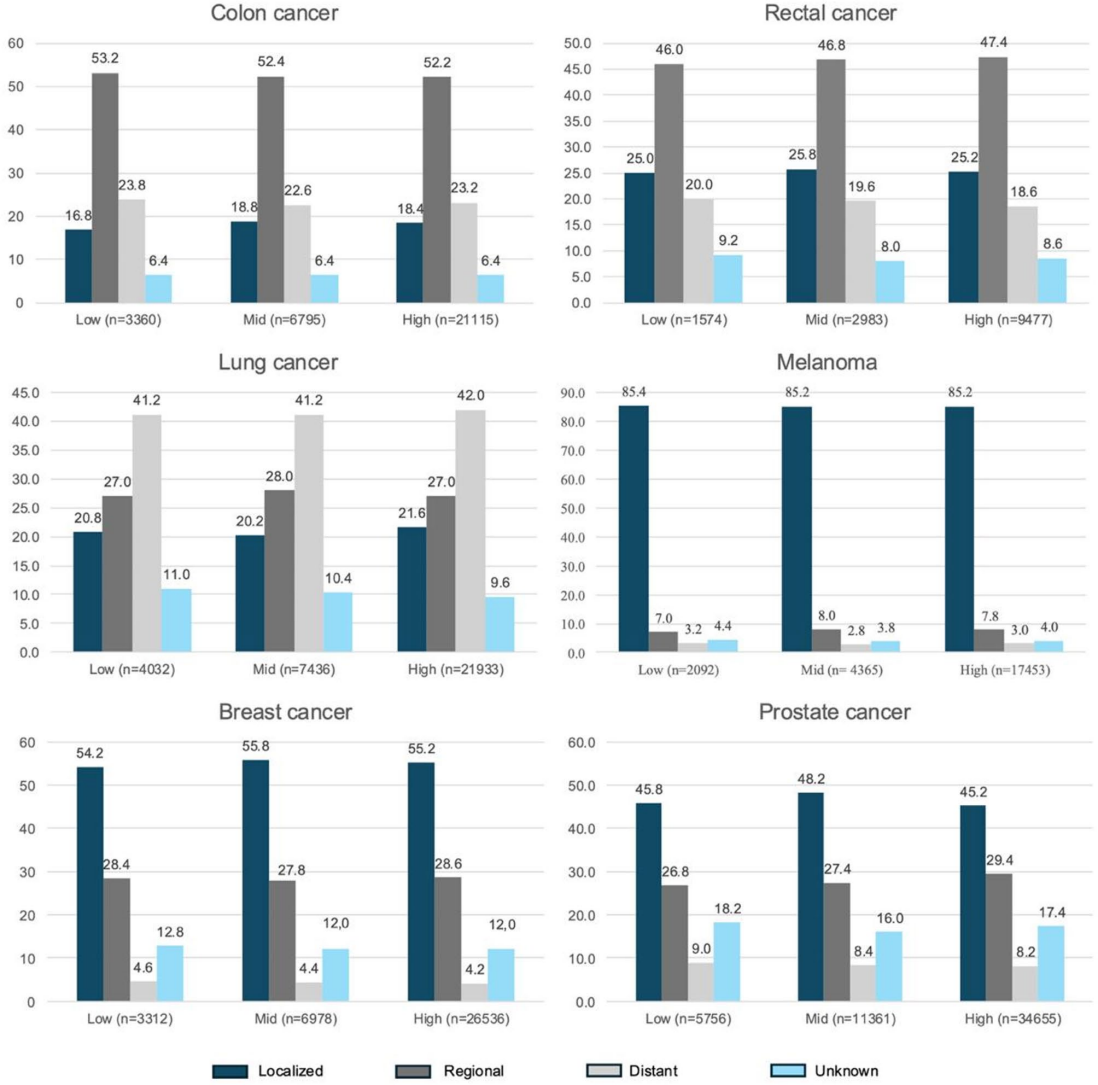
Percentage of cancer cases diagnosed at the localized, regional, distant, or unknown stage for the different cancer types from 2014–2023, categorized by educational level (low, mid, and high). Overall *p*-values for group differences and confidence intervals are reported in Online Resource 1

As shown in Fig. 1, low-education areas had a slightly higher proportion of patients diagnosed with distant metastases compared to the mid- and high-education areas for all cancer types except for lung cancer. Within this group, the rates were 23.8% for colon cancer, 20.0% for rectal cancer, 3.2% for melanoma, 4.6% for breast cancer, and 9.0% for prostate cancer. Comparatively, in the high-education areas, the proportions diagnosed with distant metastases were slightly lower: 23.2% for colon cancer, 18.6% for rectal cancer, 3.0% for melanoma, 4.2% for breast cancer, and 8.2% for prostate cancer.

At the same time, the proportion of patients diagnosed at a localized stage was generally higher in the mid- or high-education areas, as seen in colon cancer (18.4% in the high-education areas) and breast cancer (55.8% in the mid-education areas). However, we observed an exception for melanoma, where the low-education areas had the highest proportion of patients diagnosed at a localized stage.

Regional spread shows mixed trends, peaking in those with medium or high education, except for colon cancer, which was highest in the low-education areas.

Statistical testing indicated that education was significantly associated with stage at diagnosis only for lung cancer (*p* = 0.003) and prostate cancer (*p* < 0.001), whereas the differences observed for colon, rectal, melanoma, and breast cancer were not significant.

### Survival

**Fig. 2:**
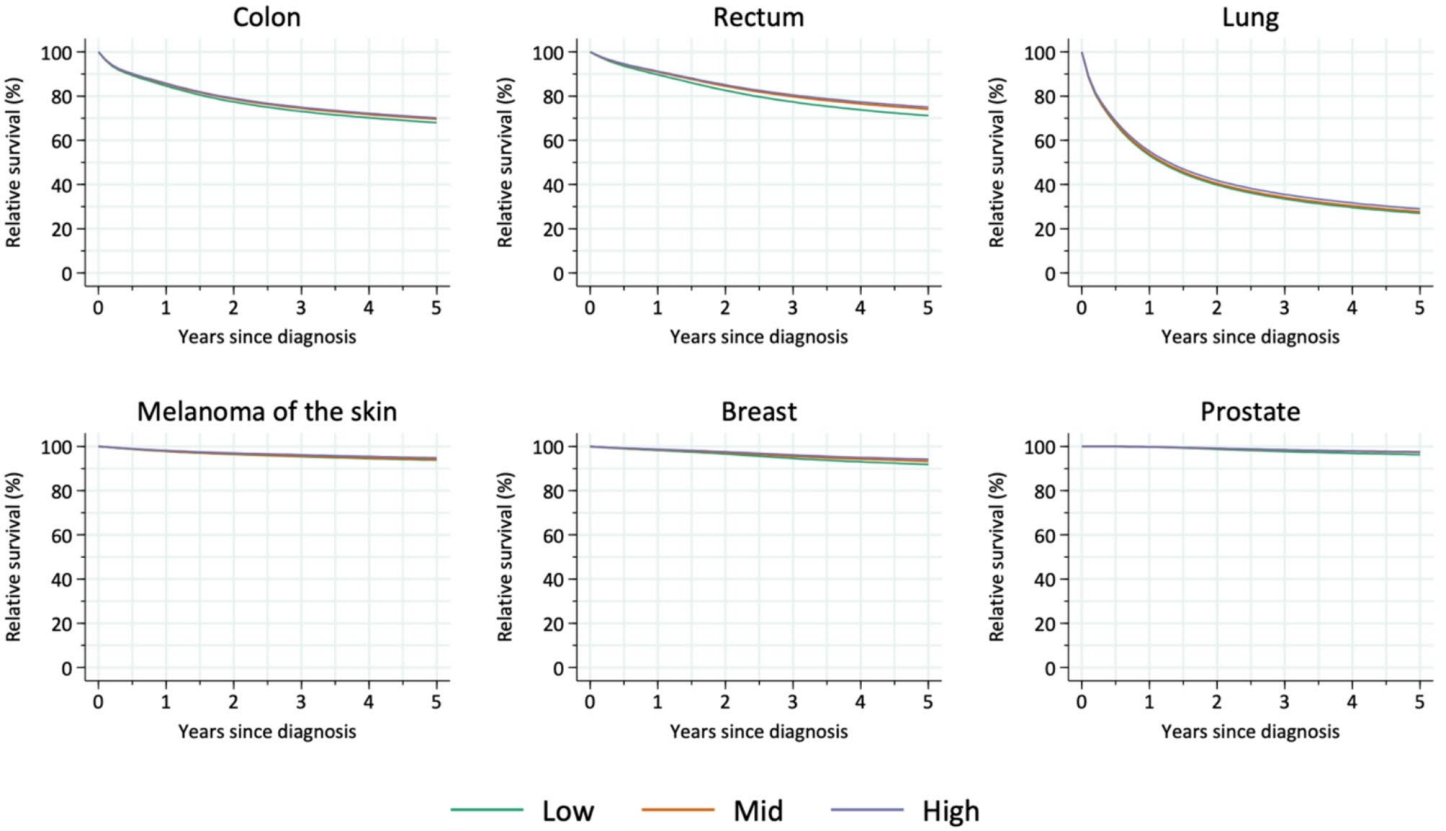
Unadjusted relative survival up to five years after diagnosis for colon, rectal, lung, melanoma of the skin, breast, and prostate cancer, stratified by educational level (low, mid, high)

The results show only minor differences in five-year relative survival between educational areas for most cancer types (Table 3; Fig. 2). The high-education areas generally had slightly higher survival rates. When adjusted for age, sex, urban/rural residence, and stage, the only cancer with a significant association between education and survival was breast cancer (*p* = 0.037).

**Table 3:** Five-year relative survival (95% confidence interval in parentheses) by educational level, unadjusted and adjusted for age, sex, urban/rural residence, and stage at diagnosis. Municipalities were grouped by educational level (low, medium, high) based on the percentage of the population with more than four years of higher education: low (≤ 5.2%), mid (5.2–6.9%), high (6.9–24.1%). Overall *p*-values are from likelihood ratio tests in adjusted models

Cancer type	Unadjusted five-year survival (95% CI)			Adjusted five-year survival			*p*-value (adjusted)
	Low	Mid	High	Low	Mid	High	
**Colon**	68(66-70.2)	69.6(68.1–71.1)	70.1(69.3–71)	69.3(67.8–70.9)	69.9(68.8–71.1)	70(69.3–70.7)	0,746
**Rectal**	71.2(68.2–74.2)	74.1(72-76.3)	75(73.8–76.2)	73.7(71.5–76.1)	73.9(72.1–75.6)	75.2(74.1–76.3)	0,355
**Lung**	27(25.5–28.6)	27.7(26.5–28.9)	29(28.2–29.7)	27.6(26.5–28.9)	28(27.1–29)	28.8(28.2–29.4)	0,175
**Melanoma of the skin**	93.8(92.1–95.5)	94.1(93-95.3)	94.9(94.3–95.4)	94.2(92.9–95.4)	94.5(93.6–95.3)	94.9(94.4–95.4)	0,518
**Breast**	91.9(90.6–93.2)	93.3(92.5–94.2)	94.2(93.8–94.6)	91.4(90.2–92.6)	92.4(91.5–93.2)	93(92.5–93.4)	0,037
**Prostate**	96.3(95.3–97.3)	97.4(96.7–98)	97.6(97.2–98)	94.4(93.8–95.1)	95(94.5–95.5)	95.1(94.8–95.4)	0,196

For colon cancer, the adjusted five-year survival was 69.6% in the low-education areas compared to 70.9% in the high-education areas. For rectal cancer, survival was 73.7% in the low-education areas and 75.0% in the high-education areas.

Lung cancer patients had a survival rate of 27.6% in the low-education areas and 28.8% in the high-education areas. For melanoma of the skin, the adjusted five-year survival was 94.2% in the low-education areas and 94.9% in the high-education areas.

For prostate cancer, survival was 94.4% in the low-education areas and 95.1% in the high-education areas.

Breast cancer patients had survival rates of 91.4% in the low-education areas and 93.0% in the high-education areas. This difference between the educational groups was statistically significant (*p* = 0.037). The likelihood ratio test showed that education contributed significantly to survival differences (*p* = 0.037), even after adjusting for age, sex, urban/rural residence, and stage at diagnosis.

## Discussion

This study indicates that educational disparities in cancer incidence vary by cancer site. Melanoma and breast cancer have a higher incidence in high-education areas, while lung cancer shows a lower incidence in high-education areas. Colon, rectal, and prostate cancer rates show small absolute differences across education groups were small, yet statistically significant. Educational differences in stage at diagnosis and survival were more consistent across the different cancer sites, with generally worse outcomes in low-education areas. Although the differences were not statistically significant for most cancer types, data analysis over a 10-year period underscores their consistency. The findings highlight a consistent trend of lower survival rates for patients in low-education areas, underscoring the potential impact of educational level on cancer outcomes. Overall, the patterns observed in incidence, stage at diagnosis, and survival were consistent with those reported in the Oslo study.

This study showed a higher incidence rate of breast cancer and melanoma in high-education areas, although the difference for breast cancer was not statistically significant. The same patterns have been reported in previous studies [7, 20], and studies focusing on individual-level data also show similar patterns [6, 30]. Several factors may explain the observed variation in breast cancer incidence. Research shows that women with higher education tend to have fewer children and are older at their first pregnancy [31]. Both factors are known to increase the risk of breast cancer [32, 33]. A meta-analysis of cohort studies showed a correlation between education level, increased alcohol consumption, and use of hormone therapy, both of which are potential risk factors for breast cancer [34].

Furthermore, studies have also shown that patients with lower educational levels have lower adherence to breast cancer screening [35, 36]. For melanoma, studies have shown a correlation between higher educational level and sun exposure [37], a known risk factor for melanoma [38]. Based on previous studies, we also assumed that the high-education areas would have a higher incidence of prostate cancer [20, 39]. However, this was not found.

Previous studies have also found similar incidence rates of colon and rectal cancer between different educational levels [20, 39]. For lung cancer, incidence was highest in low-education areas, and similar patterns have been found in earlier studies [20, 39–42]. One explanation for this may be the correlation between smoking and lower educational attainment [43, 44].

Furthermore, previous studies have indicated that lower socioeconomic status correlates with later diagnosis stages [20, 45]. This study found a similar pattern, with an exception for melanoma. Significant differences by education were found only for lung cancer (*p* = 0.003) and prostate cancer (*p* < 0.001). There are multiple potential explanations for this. One factor contributing to this pattern may be that limited health literacy also follows a social gradient, with a correlation between lower health literacy and lower education [17]. If health systems fail to provide clear and accessible information, individuals with lower health literacy may struggle to navigate the healthcare system, follow treatments or preventive measures effectively, and make informed decisions about their health [17]. For example, it may lead to difficulties in recognizing early symptoms of cancer or having knowledge of screening programs.

Lastly, we also found lower survival for all cancer types in the low-education areas, similar to findings from previous studies [20, 46, 47]. Even cancer types with a higher incidence in the high-education areas had lower survival rates when diagnosed in low-education areas, and this pattern remained after adjusting for age, sex, urban/rural residence, and stage at diagnosis. Education had a statistically significant independent effect on breast cancer survival. This disparity in survival may partly be explained by the later stage at diagnosis observed in low-education areas both in this study and the Oslo study [20]. Structural and logistical barriers may also exist, such as longer distances to hospitals and primary care physicians in low-education areas [48, 49].

The patterns observed in this study may also be explained by the fact that education was measured at the municipal level rather than the individual level. One might speculate that cancer outcomes are shaped by the community’s educational profile, not just a person’s own education. For example, people with low education in municipalities with high educational attainment may benefit from greater availability of preventive services. They may also benefit from stronger social norms around healthcare use. A previous Norwegian study found that patients who live in a municipality where the average educational level is high have a better cancer prognosis than others [50]. Another study found that, in the largest municipalities, higher average neighborhood education was significantly associated with lower individual all-cause mortality, even after accounting for each person’s own education [51]. This indicates that not only individual educational attainment matters for health outcomes, but also the broader educational level of the community. Our findings likely reflect a combination of individual education and the educational level of the community.

Overall, this study found that municipalities with lower educational attainment consistently had lower survival rates for the six most prevalent cancer types in Norway, regardless of cancer incidence. It also showed that low-education municipalities had the highest proportion diagnosed at advanced stages for melanoma of the skin, colon, rectal, breast, and prostate cancer. This study corresponds with the findings in the Oslo study, providing further evidence of education-related disparities in cancer outcomes. Similar patterns were observed regarding cancer incidence, stage at diagnosis, and survival both in Oslo and Norway. This consistency across Norway suggests that these disparities are not isolated to urban areas like Oslo and may indicate systemic issues within healthcare accessibility and education-related inequities nationwide.

### Strengths and limitations

This study is strengthened by the near-complete (98.7%) nationwide coverage of cancer diagnoses from the Cancer Registry of Norway. However, these findings should be interpreted in light of their limitations.

Since we used aggregated data, we could not include information about comorbidities, transport time to the nearest hospital or primary care physician, or other factors that may influence cancer outcomes. This may have affected the results for incidence, stage, and survival. The study identifies correlations, but several potential confounding variables could not be controlled for. Data aggregation can also mask individual-level variations and potentially lead to loss of important information. For example, while a municipality may have a high average educational level, individuals within that municipality with lower education may still experience poorer health outcomes. We did not have access to data on individual-level education due to data protection restrictions. Therefore, we cannot separate the effect of living in a high- or low-education municipality from the effect of an individual’s own education. This may complicate the interpretation of the results. Although aggregate data carries the risk of ecological fallacy and cannot fully account for within-municipality heterogeneity, it provides relevant insights into community-level disparities. Examining both the individual and aggregate level in future studies could provide a more complete understanding of educational differences in cancer outcomes.

Focusing on a recent period ensures consistency in cancer treatment protocols and minimizes random year-to-year variations. However, it limits our ability to capture long-term trends in cancer outcomes. Given that data on educational level have been available since 1970, an analysis over a longer period could provide valuable insights into how social inequalities in cancer have evolved.

### Implications and interpretations

This study highlights the correlation between educational level and cancer outcomes. Our findings are similar to those of a previous study conducted in Oslo, which showed that the low-education areas had higher lung cancer incidence, lower five-year relative survival rates overall, and a greater proportion of distant-stage diagnoses for most cancer types. This similarity highlights the consistency of the results in the current study. The association between lower educational level, a higher proportion diagnosed at late stages, and poorer survival outcomes suggest possible barriers faced by those in the low-education areas. These barriers may include limited access to healthcare, lower health literacy, and/or challenges in accessing or utilizing preventive care and cancer screening services. Higher education was linked to earlier diagnoses and better survival rates, possibly reflecting greater health awareness, better health-seeking behavior, and easier access to healthcare. The variations in cancer incidence may suggest differences in risk factors, genetic predisposition, and lifestyle.

Addressing cancer disparities will require public health strategies that tackle the root causes of the inequalities. For instance, underserved communities with lower educational attainment may benefit from better access to cancer screening, preventive care, and health education campaigns. Disparities can also be maintained, enforced or reduced at the clinical level. However, the evidence base remains insufficient for reliable decision-making. Future research should focus on uncovering factors that perpetuate these disparities, such as differences in healthcare infrastructure and cultural influence on health-seeking behavior.

## Supplementary Information

Below is the link to the electronic supplementary material.

Supplementary Material 1

## Data Availability

Data underlying this article can be requested from the Cancer Registry of Norway through https://helsedata.no.
