# Supplementary material for: Educational differences in cancer incidence, stage at time of diagnosis, and survival in Norway
Source: Res Health Serv Reg. 2025 Oct 3;4:15. doi: 10.1007/s43999-025-00075-z (PMC12491118; doi:10.1007/s43999-025-00075-z)
Supplement: Supplementary file 1 — Supplementary Material 1 [file 43999_2025_75_MOESM1_ESM.docx]

Educational differences in cancer incidence, stage at time of diagnosis, and survival in Norway.

Kenz Al-Shather ^1,2^*, Yngvar Nilssen ^3^ , Inger Kristin Larsen^3^ , Erlend Hem ^1,2^ and Berit Horn Bringedal ^2^.

*Correspondence:

Kenz Al-Shather

[KenzAlShather@gmail.com](mailto:KenzAlShather@gmail.com)

ORCID: 0009-0006-9339-5997

1. Department of Behavioural Medicine, Institute of Basic Medical Sciences, Faculty of Medicine, University of Oslo, Oslo, Norway 2
2. Institute for Studies of the Medical Profession, PO Box 1152, NO‑0107 Sentrum, Oslo, Norway 3
3. Department of Registration, Cancer Registry of Norway, Oslo, Norway.

| **Cancer type** | | **Educational level** | | | **Overall p-value** |
| --- | --- | --- | --- | --- | --- |
| **Colon Cancer** | | | | | **p = 0.34** |
|  | | **Low (n=3360)** | **Mid (n=6795)** | **High (n=21115)** |  |
| Stage at diagnosis | Localized | 16·8% (4·8 – 75·0) | 18·8% (4·8 – 50·0) | 18·4% (6·3 – 100) |  |
|  | Regional | 53·2% (15·4 – 80·0) | 52·4% (16·7 – 81·0) | 52·2% (33·3 – 87·5) |  |
|  | Distant | 23·8% (5·6 – 66·7) | 22·6% (4·8 – 46·2) | 23·2% (6·7 – 53·3) |  |
|  | Unknown | 6·4% ( 2·2 – 26·7) | 6·4% (2·0 – 33·3) | 6·4% (1·4 – 33·3) |  |
| **Rectal Cancer** | | | | | **p = 0.60** |
|  | | **Low (n=1574)** | **Mid (n=2983)** | **High (n=9477)** |  |
| Stage at diagnosis | Localized | 25·0% (5·0 – 100) | 25·8% (8·3 – 75·0) | 25·2% (10·0 – 100) |  |
|  | Regional | 46·0% (14·3 – 100) | 46·8% (12·5 – 100) | 47·4% (11·1 – 100) |  |
|  | Distant | 20·0% (4·8 – 75) | 19·6% (4·2 – 80·0) | 18·6% (6·7 – 66·7) |  |
|  | Unknown | 9·2% (4·3 – 50·0) | 8% (2·1 – 100) | 8·6% (1·1 – 41·2) |  |
| **Lung Cancer** | | | | | **p = 0.003** |
|  | | **Low (n=4032)** | **Mid (n=7436)** | **High (n=21933)** |  |
| Stage at diagnosis | Localized | 20·8% (6·7 – 50) | 20·2% (4·0 – 37·1) | 21·6% (6·3 – 42·9) |  |
|  | Regional | 27·0% (7·7 – 80·0) | 28·0% (9·1 – 60·0) | 27·0% (14·3 – 60·0) |  |
|  | Distant | 41·2% (9·1 – 100) | 41·2% (17·6 – 100·0) | 42% (14·3 – 85·7) |  |
|  | Unknown | 11·0% (1·8 – 50·0) | 10·4% (1·8 – 45·5) | 9·6% (1·2 – 28·6) |  |
| **Melanoma** | | | | | **p = 0.77** |
|  | | **Low (n=2092)** | **Mid (n= 4365)** | **High (n=17453)** |  |
| Stage at diagnosis | Localized | 85·4% (33·3 – 100) | 85·2% (40·0 – 100) | 85·2% (50·0 – 100) |  |
|  | Regional | 7·0% (3·1 – 66·7) | 8·0% (1·8 – 33·3) | 7·8% (2·0 – 50·0) |  |
|  | Distant | 3·2% (1·2 – 25) | 2·8% (0·7 – 28·6) | 3% (1·0 – 20·0) |  |
|  | Unknown | 4·4% (1·7 – 33·3) | 3·8% (1·7 – 33·3) | 4% (1·1 – 50·0) |  |
| **Breast Cancer** | | | | | **p = 0.58** |
|  | | **Low (n=3312)** | **Mid (n=6978)** | **High (n=26536)** |  |
| Stage at diagnosis | Localized | 54·2% (20·0 – 100) | 55·8% (33·3 – 100) | 55·2% (23·1 – 100) |  |
|  | Regional | 28·4% (12·5 – 75·0) | 27·8% (7·7 – 66·7) | 28·6% (14·8 – 58·8) |  |
|  | Distant | 4·6% (1·6 – 40·0) | 4·4% (1·4 – 33·3) | 4·2% (1·4 – 11·8) |  |
|  | Unknown | 12·8% (2·2 – 60·0) | 12·0% (3·2 – 66·7) | 12·0% (3·4 – 46·2) |  |
| **Prostate Cancer** | | | | | **p < 0.001** |
|  | | **Low (n=5756)** | **Mid (n=11361)** | **High (n=34655)** |  |
| Stage at diagnosis | Localized | 45·8% (16·7 – 78·4) | 48·2% (14·3 – 71·2) | 45·2% (25·0 – 100) |  |
|  | Regional | 26·8% (8·3 – 57·1) | 27·4% (10·1 – 52·4) | 29·4% (5·6 – 50·0) |  |
|  | Distant | 9·0% (2·3 – 50·0) | 8·4% (2·9 – 28·6) | 8·2% (1·1 – 25·0) |  |
|  | Unknown | 18·2% (2·7 – 50·0) | 16·0% (4·0 – 36·4) | 17·4% (4·2 – 40) |  |

**Percentage of cancer cases diagnosed at the localized, regional, distant, or unknown stage for the different cancer types from 2014-2023, categorized by educational level (range in parentheses). Differences in stage at diagnosis across educational groups were assessed using Pearson’s chi-square test.**
